# Why precision medicine is not the best route to a healthier world

**DOI:** 10.11606/S1518-8787.2018052000209

**Published:** 2018-01-29

**Authors:** Juan Pablo Rey-López, Thiago Herick de Sá, Leandro Fórnias Machado de Rezende

**Affiliations:** I University of Sydney. School of Public Health. Prevention Research Collaboration. Sydney, NSW, Australia; II Universidade de São Paulo. Núcleo de Pesquisas Epidemiológicas em Nutrição e Saúde. São Paulo, SP, Brasil; III Universidade de São Paulo. Faculdade de Medicina. Departamento de Medicina Preventiva. São Paulo, São Paulo, Brasil

**Keywords:** Public Health, Precision Medicine, Therapeutic Approaches, Risk Factors, Preventive Medicine

## Abstract

Precision medicine has been announced as a new health revolution. The term precision implies more accuracy in healthcare and prevention of diseases, which could yield substantial cost savings. However, scientific debate about precision medicine is needed to avoid wasting economic resources and hype. In this commentary, we express the reasons why precision medicine cannot be a health revolution for population health. Advocates of precision medicine neglect the limitations of individual-centred, high-risk strategies (reduced population health impact) and the current crisis of evidence-based medicine. Overrated “precision medicine” promises may be serving vested interests, by dictating priorities in the research agenda and justifying the exorbitant healthcare expenditure in our finance-based medicine. If societies aspire to address strong risk factors for non-communicable diseases (such as air pollution, smoking, poor diets, or physical inactivity), they need less medicine and more investment in population prevention strategies.

## INTRODUCTION

Precision medicine (also referred to as personalised medicine) has been proposed as the new route to offset the gloom-and-doom forecasts for non-communicable diseases in developed countries. For example, some projections indicate that cardiovascular disease rates will rise in USA by 10% between 2010 and 2030^6^. The term “precision” implies more accuracy in healthcare and prevention of diseases, which may yield substantial health care cost savings^4^. This paradigm relies on the narrative that an ever-increasing knowledge of biological mechanisms, especially genetics, coupled with information technology will lead to transformative improvements in population health. To illustrate this, in 1999, Francis Collins envisioned a revolution in medicine with the Human Genome Project^4^. However, many of the prospects announced by precision medicine advocates have failed^2^ ^,^ ^11^ ^,^ ^12^. Epidemiologists Bayer and Galea^2^ recently made a convincing case about the limitations of precision medicine stating that health differences among different social groups are mainly not driven by better clinical treatments but by marked differences in socioeconomic factors. They have quoted the lesson learnt with the Whitehall studies of British civil servants, in which free healthcare did not avoid a marked social health gradient among groups. The case of United States is even more illustrative. Although the USA spends approximately 18% of its gross domestic product in health care (twice the rate of other rich countries), Americans attain poorer health indicators than citizens from many other developed economies^19^. Furthermore, the key idea underpinning the Human Genome Project and precision medicine (for most diseases, a reduced number of common genetic variants may predict disease risk) has been rejected and the translation from bench to bedside of the genetic knowledge is highly disappointing, as no therapies have been developed to treat a single gene disorder, such as sickle cell anemia^10^. Why, then, $15 billion of the $26 billion of extramural research funding sponsored by the National Institutes of Health (NIH) in USA is precision medicine-related research (e.g., gene, genome, stem cells, or regenerative medicine)^11^?

Here, we call for greater recognition of the social determinants of health in the biomedical community and we suggest some solutions that could help to mitigate the burden of non-communicable diseases worldwide.

## THE PRIMARY DETERMINANTS OF HEALTH ARE SOCIOPOLITICAL

It is well established that social disadvantaged groups have a lower life expectancy and quality of life (disability-free life expectancy) in both high-income and low- to middleincome countries^14^. In fact, as the evidence of the impact of social inequalities in health was robust in 2005, the World Health Organization set up the Commission on Social Determinants of Health to promote action^14^. The social gradient in health gets under the skin from the very early stages of life (for a detailed information about this topic, see the book “The Health Gap”^14^). Unfortunately, policies that specifically address socioeconomic inequalities, improve job conditions, increase the availability of healthy foods, and empower individuals to follow physically active lifestyles are virtually nonexistent. In contrast, blue-sky research receives a disproportionately large investment with a very dynamic market, but with unproven, uncertain returns in terms of population health. Furthermore, under free market rules, “evidence”-based medicine serves different commercial agendas than originally intended^8^. Next, we illustrate with some examples the limited public health impact of precision medicine.

## WHY INDIVIDUAL CENTRED ATTEMPTS TO CHANGE HEALTH BEHAVIOURS ARE INEFFECTIVE

Advocates of precision medicine claim that advances and implementation of new technologies will optimize prevention strategies^13^. However, regardless of how technologically sophisticated a society may evolve, citizens should be very careful with all these promises, because:

1. An excessive emphasis in technological solutions to prevent or treat diseases diverts our attention from the root of the problem: health relies on favourable social circumstances^14^;
2. In addition, clinical medicine and “precision” prevention have become an industry advertisement tool^8^. Protecting individuals against big corporations in the current political scenario may sound naive given the constant economic inequalities experienced in the world in the last decades^16^. In a fair world, healthy living directly threatens many powerful corporations (e.g. diet: consumption of less sugar and meat; physical activity: promotion of active transport in cities instead of cars, investment in green areas instead of real state, and promotion of a more extensive use of non-electronic activities during leisure time). Even better population health indicators would require a lower demand of healthcare professionals and pharmacological products.

The argument that technologies may help us make more sustainable healthy choices should be subject to scrutiny given the over simplistic approach of behavioural change behind such claims. For example, in a recent randomized controlled trial, the provision of one physical activity wearable monitor to increase weight loss by higher physical activity did not offer advantages compared with standard weight lost approaches without these devices^9^. Similarly, personalized nutrition advice (internet-delivered intervention) based on information on individual diet and lifestyle, phenotype, or genotype did not promote larger and sustained changes in dietary behaviour compared with non-personalized approaches (advice based on dietary guidelines)^3^. As shown in Table S3 in this study^3^, after six months of intervention, the intake of fruits, vegetables, whole grains, oily fish, red meat, low-fat dairy products, salt, and total energy intake did not differ between the control group and the pooled personalized group. Finally, evidence is growing on the fact that the communication of genetic risk of diseases to individuals, a fundamental basis of precision medicine^12^, does not lead to changes in health behaviours^7^. Taken together, the narrative that the widespread implementation of technology will help us change and sustain behaviours seems to be fake claims of precision medicine advocates. Indeed, social, cultural, environmental, and economic factors largely determine health-related behaviours^14^.

## FAIR AND EGUALITARIAN SOCIETIES: HEALTHY LIVES

In summary, precision medicine seems to be a secondary route to a healthier world. The XXI century is becoming a turning point for life on Earth – human-made climate disruption, chaotic urbanization, forced migration, pollution, degradation, and depletion of natural resources are all examples of challenges^15^ for which precision medicine has little to offer compared to broader public health approaches. The evidence supports policies that address climate change and human health simultaneously, such as the promotion of non-motorized transport^18^ or the reduction of animal sourced foods in our diets (i.e., a study has estimated that global mortality could be reduced by 6%–10% and food-related greenhouse gas emissions could be reduced by 29%–70%)^17^. Enough scientific evidence shows that too much medicine is harming humans and should therefore be abandoned. For example, although the market has many valuable drugs, the overall balance for the population indicates that their dangers outweigh their benefits^5^ and there are good reasons (e.g., over-diagnosis) to rethink the widespread screening programs for cancer prevention^1^. Advocates of the precision medicine initiative neglect the limitations of person-centred, high-risk strategies (a reduced population health impact) and, therefore, their messages may be useful to dictate priorities in the research agenda (e.g., overestimating the role of genetics, stem cell therapies) and to justify the exorbitant healthcare expenditure in the current era of finance-based medicine. If societies aspire to address strong risk factors for non-communicable diseases^8^ such as smoking, poor diets, or physical inactivity, they will require downplaying the numerous promises of precision medicine and a greater appreciation of the importance of spending more resources on the foundational conditions that shape the health of the populations.
